# Extracellular environment contribution to astrogliosis—lessons learned from a tissue engineered 3D model of the glial scar

**DOI:** 10.3389/fncel.2015.00377

**Published:** 2015-09-29

**Authors:** Daniela N. Rocha, José P. Ferraz-Nogueira, Cristina C. Barrias, João B. Relvas, Ana P. Pêgo

**Affiliations:** ^1^Instituto de Engenharia Biomédica (INEB), Universidade do PortoPorto, Portugal; ^2^Instituto de Investigação e Inovação em Saúde, Universidade do PortoPorto, Portugal; ^3^Faculdade de Engenharia, Universidade do PortoPorto, Portugal; ^4^Glia Cell Biology Group, Instituto de Biologia Celular e Molecular, Universidade do PortoPorto, Portugal; ^5^Instituto de Ciências Biomédicas Abel Salazar, Universidade do PortoPorto, Portugal

**Keywords:** astrogliosis, astrocytes, extracellular matrix, mechanical properties, mechanotransduction, RhoA

## Abstract

Glial scars are widely seen as a (bio)mechanical barrier to central nervous system regeneration. Due to the lack of a screening platform, which could allow *in-vitro* testing of several variables simultaneously, up to now no comprehensive study has addressed and clarified how different lesion microenvironment properties affect astrogliosis. Using astrocytes cultured in alginate gels and meningeal fibroblast conditioned medium, we have built a simple and reproducible 3D culture system of astrogliosis mimicking many features of the glial scar. Cells in this 3D culture model behave similarly to scar astrocytes, showing changes in gene expression (e.g., GFAP) and increased extra-cellular matrix production (chondroitin 4 sulfate and collagen), inhibiting neuronal outgrowth. This behavior being influenced by the hydrogel network properties. Astrocytic reactivity was found to be dependent on RhoA activity, and targeting RhoA using shRNA-mediated lentivirus reduced astrocytic reactivity. Further, we have shown that chemical inhibition of RhoA with ibuprofen or indirectly targeting RhoA by the induction of extracellular matrix composition modification with chondroitinase ABC, can diminish astrogliosis. Besides presenting the extracellular matrix as a key modulator of astrogliosis, this simple, controlled and reproducible 3D culture system constitutes a good scar-like system and offers great potential in future neurodegenerative mechanism studies, as well as in drug screenings envisaging the development of new therapeutic approaches to minimize the effects of the glial scar in the context of central nervous system disease.

## Introduction

Astrocytes are the most abundant cells in the central nervous system (CNS) (Lu et al., 2006) and are known to play a pivotal role in glial scar formation. Reactive astrogliosis starts when a trigger-stimulus produced at the injury site drives astrocytes to leave their quiescent state and become activated. Reactive astrocytes are characterized by increased expression of intermediate filament proteins glial fibrillary acidic protein (GFAP) and vimentin (Holley et al., 2003; Wang et al., 2004; Robel et al., 2011), augmented production of extracellular matrix (ECM) constituents, such as collagen IV (Liesi and Kauppila, 2002) and chondroitin sulfate proteoglycans (CSPG) (Busch and Silver, 2007), and increase in the production of matrix metalloproteinases (MMPs) thought to be closely associated to ECM remodeling (Ogier et al., 2005, 2006; Nair et al., 2008). The regeneration failure in the adult CNS is multi-factorial but the glial scar has been ascribed has a highly inhibitory environment. While it has been a widely explored therapeutic target (Jones et al., 2003; Koechling et al., 2011), very little is known about the causes and mechanisms underlying astrocyte activation.

Several animal models have been developed to study the processes of CNS degeneration and regeneration. Nevertheless, these are time consuming, costly, and raise technical and ethical issues when one intends to perform routine assays to elucidate molecular mechanisms or screening for potential therapeutics. These emphasize the need to develop simpler experimental systems. The existing 2D *in vitro* astrogliosis models have provided important insights (Wanner et al., 2008; Kimura-Kuroda et al., 2010; Koechling et al., 2011) but they do not replicate key distinctive features of the ECM in a glial scar. As such, the development of a 3D model would be of added value, as this can better recapitulate several features of native cellular microenvironments, by incorporating both biochemical and mechanical components. The biggest challenge is to recreate simple, yet biologically meaningful matrices that support cells within the lesion environment, with a minimum number of model system variables. ECM-derived natural matrices such as Matrigel® or decellularized tissue provide factors that support cell function; however, the inherent complexity and variability of these scaffolds makes it difficult to isolate and dissect cell-signaling mechanisms (Owen and Shoichet, 2010). Here, a new *in vitro* alginate based 3D model of the glial scar is proposed to serve as a tool in the identification and modulation of molecular mechanisms underlying astrocyte activation. Mammalian cells do not interact with alginate, therefore it constitutes a relatively inert backbone structure (Rowley et al., 1999; Lutolf and Blau, 2009). Moreover, alginate based matrices are highly reproducible, a pivotal requirement for their application as 3D artificial ECM.

Cerebral astrocytes were cultured within 3D alginate discs with different alginate contents, and consequently different mechanical properties. These were further stimulated with conditioned medium from meningeal fibroblasts, in order to mimic the possible stimuli resultant from fibroblast infiltration occurring following CNS injury. Mechanical properties of CNS tissue are known to be altered when a glial scar is formed (Bonneh-Barkay and Wiley, 2009; Freimann et al., 2011; Murphy et al., 2012) and ECM components are thought to play a pivotal role on the mechanotransduction processes in healthy and diseased tissues. The correlation between astrocyte reactivity, ECM production and composition and the mechanical properties of the surrounding environment was explored. We show that the Rho-ROCK signaling pathway can regulate astrogliosis constituting a possible therapeutic target.

## Materials and methods

Unless mentioned otherwise all reagents were supplied by GIBCO and were of cell culture grade.

### Animals

Procedures involving animals and their care were conducted in compliance with institutional ethical guidelines (IBMC) and with the approval of Portuguese Veterinary Authorities. Animals had free access to food and water, being kept under a 12-h light/ 12-h dark cycle.

### Cell isolation

#### Meningeal fibroblasts and cerebral astrocytes

Meningeal fibroblasts and astrocytes were obtained as previously described (Kimura-Kuroda et al., 2010). Briefly, meningeal fibroblasts were obtained from brain meninges of P2 Wistar Han rats. Upon isolation, meningeal tissue was digested in Hank's Balanced Salt Solution (HBSS) without calcium or magnesium, supplemented with papain (20 U/mL, Sigma-Aldrich), for 30 min. Dissociated meninges were plated in poly-L-lysine (Sigma-Aldrich) coated 75 cm^2^ flasks (BioLite), and maintained in Dulbecco's Modified Eagle Medium (DMEM) supplemented with 10% (v/v) inactivated fetal bovine serum (FBS) and 1% (v/v) penicillin-streptomycin (PS).

Fibroblast conditioned medium (CM) was obtained by culturing 13.3 cells.cm^−2^ in DMEM supplemented with 10% FBS and 1% PS, for 72 h. After collection, CM was centrifuged and stored at 4°C until use.

Cerebral cortices were further dissected, after removal of the meninges. Isolated cortices were digested in HBSS without calcium or magnesium supplemented with papain (0.2 U/ml), for 30 min. Dissociated cortices were cultured in 75 cm^2^ flasks and maintained in DMEM supplemented with 10% (v/v) FBS and 1% (v/v) PS. When confluence was reached (~12 days) the flasks were shaken overnight on an orbital shaker (240 rpm) at 37°C to remove loosely attached microglia, oligodendrocytes and neurons. The remaining cells, mainly astrocytes, adhered to the 75 cm^2^ flasks were then trypsinized and cultured in new flasks. Further, tripsinizations were performed in order to increase culture purity.

Mice astrocytes were isolated from P1 flox RhoA mice. After meninges were removed, isolated cortices were digested for 30 min in HBSS without calcium or magnesium supplemented with papain (0.2 U/ml). Dissociated cortices were cultured in 75 cm^2^ flasks and maintained in DMEM supplemented with 10% (v/v) horse serum and 1% (v/v) PS. Cells were cultured until confluence was achieved. Astrocyte purification was achieved as previously described.

#### Cortical neurons isolation and co-culture with astrocytes

To obtain cortical neurons, E18 Wistar Han rat embryos were recovered by cesarean section of pregnant rats. The isolated cortices were dissociated for 30 min at 37°C in HBSS supplemented with 1 mM pyruvate, 2 mg.ml^−1^ albumin, and 10% (v/v) trypsin. Viable cells (trypan blue exclusion assay) were seeded at a density of 2.2 × 10^4^ viable cells/cm^2^ in DMEM:Nutrient Mixture F-12 (3:1) supplemented with 10% (v/v) inactivated fetal calf serum. Two hours later, medium was changed to Neurobasal medium supplemented with 0.5 mM L-glutamine, 2% (v/v) B27 supplement, 1% (v/v) PS and 0.5% (v/v) Gentamicin. For the co-culture, astrocytes were cultured for 4 days, in DMEM supplemented with 10% FBS and 1% PS, prior to cortical neurons culture. At day 4 the DMEM culture medium was removed and cortical neurons were seeded on top of the adherent astrocytes. The co-culture were maintained for 4 additional days and then fixed with 4% paraformaldehyde in phosphate buffered saline (PBS).

#### Alginate discs preparation

*In situ* forming alginate hydrogel matrices were prepared as previously described (Maia et al., 2014). Briefly, PRONOVA ultrapure sodium alginates LVG and VLVG (hereafter designated as high and low molecular weight, HMW and LMW, respectively) with a high guluronic acid content (68%) were purchased from FMC Biopolymers. Hydrogel-precursor solutions with a bimodal molecular weight composition were prepared by combining HMW and LMW alginate at a 1:1 volume ratio and at different total polymer concentrations (0.5, 1, and of 2% w/v). Primary rat astrocytes were added to alginate solutions (4 × 10^6^ cells.mL^−1^) with CaCO_3_ (Ca^2+^/COO^−^ molar ratio = 0.288) and δ-gluconolactone (GDL, Ca^2+^/GDL molar ratio = 0.125), and the mixture was pipetted (20 μL) onto the wells of pHEMA-treated culture plates. After crosslinking (1 h, 37°C), cell-laden 3D matrices were maintained in culture for 7 days, in DMEM or CM.

Ibuprofen (0.04 M) and chondroitinase ABC (chABC) (0.1 U.mL^−1^) were added to the 3D cultures at day 7 of culture, and were maintained for additional 48 h.

#### ATP quantification

ATP quantification was performed using the CellTiter-Glo Luminescent Cell Viability Assay (Promega) according to the manufacturer's recommendations. Briefly, the CellTiter-Glo® Reagent was added directly to cells cultured in serum-supplemented medium. This resulted in cell lysis and generation of a luminescent signal proportional to the amount of ATP present, which was measured in a luminescent plate reader (SYNERGY MX, BioTek). An ATP standard curve was performed using ATP disodium salt hydrate (Sigma).

#### Cell viability

At culture days 1, 3, and 7 alginate discs were incubated with a solution of calcein-AM (Promega) for 20 min, followed by 5 min incubation with propidium iodide (Sigma). Discs were rinsed in culture medium twice to wash any excess of calcein-AM and propidium iodide and finally observed under the confocal microscope.

For flow cytometry analysis, cells were firstly incubated with a 6 μM solution of propidium iodide (PI, Sigma-Aldrich) for 10 min at 37°C. Cells were further extracted from the alginate disks using trypsin-EDTA and transferred to 96-well round bottom plates and washed with 150 μL of FACS Buffer (2% FBS in PBS 1X) by centrifuging for 3 min, 244 g at RT. For data acquisition, cells were suspended in 150 μL of FACS Buffer and analyzed on a BD FACS Canto II cytometer using 530/30 and 670/LP optical filters. The cell population of interest was gated according to forward (FSC), side scatter (SSC) and fluorescence parameters using untreated cells. Doublets were excluded with FSC-peak (height) vs. FSC-integral (area) gating. A total of 20 000 events were acquired per sample. Data was analyzed using FlowJo software version vX.0.7.

#### Neurite outgrowth quantification and cell motility

For axonal outgrowth assessment the length of the longest neurite was determined using AxioVision image analysis software. Neuronal processes were manually traced and quantified on a total of 95 cells per condition from 3 different samples. Cell motility was assessed using ImageJ software with the MTrackJ pluggin. The motility profile was traced for 30 cells per condition from 3 different samples.

#### Astrocyte infection

HEK293T cells at 80% confluence were co-transfected with JetPrime (PolyPlus Transfection) according to the manufacturer's instructions. Transfection ratios were as follows: 3 mg of shRNA plasmids to 4.2 mg of psPAX2 to 2.7 mg of Vesicular Stomatitis Virus Glycoprotein (VSVG). Medium was replaced 4 h after transfection, and cells were cultured for additional 48 h. Medium with viral particles was then collected and centrifuged. Finally, supernatants containing viral particles were collected.

Infection of primary astrocytes was performed at 80% confluence with viral supernatants overnight. Infection medium was then replaced by fresh medium with puromycin. Cells were kept in culture for 7–12 days.

#### Gene expression analysis

Cell lysis and RNA purification were performed using Quick-RNA MiniPrep from Zymo Resarch, according to the manufacturer's instructions. Reverse transcription was done with SuperScript III (Invitrogen).

#### RT-PCR

Primer sequences used for RT-PCR were as follows:

Gfap sense 5′AGGCTGGAGGCGGAGAAC3′;
Gfap anti-sense 5′GCTGTGAGGTCTGGCTTGG3′;
Vimentin sense 5′CGTGATGTCCGCCAGCAGTATG3′;
Vimentin anti-sense 5′GGCATCCACTTCGCAGGTGAG3′;
Collagen IV sense 5′AAGGCGAGGAAGGCATCATG3′;
Collagen IV anti-sense 5′GGGTGAGTAGGCTGGAGGTC3′;
Hprt sense 5′ATGGACTGATTATGGACAGGACTG3′;
Hprt anti-sense 5′GCAGGTCAGCAAAGAACTTATAGC3′.

PCR was performed using HotStarTaq DNA polymerase (Quiagen) for 34 cycles. Quantification of band intensity was done using ImageLab software.

#### Quantitative RT-PCR

Quantitative real-time PCR (qPCR) was performed using Hprt as endogenous control to normalize the expression levels of the genes of interest. Analyses were performed on iG5 (Bio-Rad) using SYBR Green (SYBR Green master mix, Applied Biosystems) according to the manufacturer's recommendations. Reactions were carried out in triplicate (40 cycles). In order to verify the specificity of the amplification, a melt-curve analysis was performed immediately after the amplification protocol. Non-specific products of PCR were not found in any case. Primer sequences used for qRT-PCR were as follows:

Gfap sense 5′GCGGCTCTGAGAGAGATTCG3′;
Gfap anti-sense 5′TGCAAACTTGGACCGATACCA3′;
RhoA sense 5′TCAGCAAGGACCAGTTCCCAGAGG3′;
RhoA anti-sense 5′AGGCCGCAGGCGGTCATAATCTTC3′;
RhoB sense 5′ TTTGCTCTGCACAGAGAATG3′;
RhoB anti-sense 5′TGGTAAAGGAAGGCAACACG3′;
RhoC sense 5′ TAGCCAAAGGCACTGATCCT3′;
RhoC anti-sense 5′GCATACCAGGAGAGAGCTGG3′;
ROCK1 sense 5′CGAGAGTGTGACTGGTGGTC3′;
ROCK1 anti-sense 5′CTGGTGCTACAGTGTCTCGG3′;
Src sense 5′GGACAGTGGCGGATTCTA3′;
Src anti-sense 5′GGTAGTGAGACGGTGACA3′;
Hprt sense 5′ATGGACTGATTATGGACAGGACTG3′;
Hprt anti-sense 5′GCAGGTCAGCAAAGAACTTATAGC3′.

#### Rheological analysis

Rheological measurements were carried out using a Kinexus Pro rheometer from Malvern with parallel-plate geometry with sandblaster surfaces, at 37°C and with 10% of compression. First, the linear viscoelastic region was analyzed for all the samples by performing a stress sweep at constant frequency of 0.1 Hz. Frequency sweeps in the linear viscoelastic regimen were used to determine values of elastic (G′) and viscous (G″) modulus. Samples were analyzed at day 1 and 7 of culture (*n* = 3 for each).

#### Gelatin zymography

Cells were switched to serum-free conditions for 24 h. After 24 h, cell culture supernatants were collected and kept at −20°C until use. MMP-2 activity was analyzed by gel zymography. Zymography was performed using a 10% SDS-Page separating gel with 0.1% gelatin (Sigma). After running, the gels were incubated in re-naturation buffer (2% triton X-100) for 30 min, with soft agitation. Then zymogram gels were changed to a development buffer (50 mM Tris-HCL, 10 mM CaCl_2_) overnight at 37°C. Afterwards gels were stained with Comassie Blue for 20 min and finally de-stained with water. Band intensity was quantified using a densitometer (Bio-Rad) and quantity one software.

#### Collagen quantification

Collagen quantification was performed with the Sircol Collagen Assay (Biocolor), according to the manufacturer's recommendations. Briefly, collagen from samples was precipitated with Sircol dye and further dissolved. Colorimetric alterations were measured at 555 nm and results were quantified using a standard curve for collagen.

#### Immunocytochemistry

2D cultured cells were fixed with 4% (v/v) paraformaldehyde. 3D cultured cells were fixed as in 2D, but CaCl_2_ was added to the solution to keep hydrogel disc integrity. Cells were further permeabilized and blocked in phosphate buffered saline (PBS), or instead in tris buffered saline with calcium chloride (TBS-CaCl_2_) for 3D discs, containing 5% (v/v). Normal Goat Serum (NGS) (Biosource) and 0.2 % (v/v) Triton X-100 (Sigma). Primary antibodies were diluted in PBS or TBS-CaCl_2_ containing 1% (v/v) NGS and 0.15% (v/v) Triton X-100, and incubated overnight in a humid chamber at 4°C. The following primary antibodies were used: rabbit anti-GFAP (1:500, Dako), mouse anti-vimentin (1:100, ThermoScientific), mouse anti-NG2 (1:100, Abcam), rat anti-MBP (1:500, AbD Serotec) rabbit anti-TAU (1:100, Sigma), mouse anti-CSPG (1:200, Millipore). Secondary antibodies Alexa-Fluor 488, 568, 594, and 660 were applied for 1 h at RT and subsequently treated for nuclear counterstaining at RT with Hoechst (Molecular Probes) at 2 μl.ml^−1^. 3D samples were then observed under confocal microscope.

#### Western blot

Cells were washed with PBS and lysed in lysis buffer (1 mM sodium orthovanadate, protease inhibitor cocktail (Amersham), 50 mM TRIS, 1% (v/v) nonyl phenoxypolyethoxylethanol (v/v), 0.5% (wt/v) sodium deoxycholate, 0.1% (wt/v) sodium dodecyl sulfate. Protein lysates (30 μg/lane) were run on a 12% SDS-Page gel and then transferred to nitrocellulose membranes (Amersham). For Western blot analysis, membranes were blocked with blocking buffer (5% (w/v) non-fat dried milk in tris-buffered saline (TBS) plus 0.1% (v/v) Tween 20) and incubated overnight at 4°C in 5% (w/v) bovine serum albumin (BSA) in TBS plus 0.1% Tween 20 with primary antibodies. The following primary antibodies were used: mouse anti-GAPDH (1:10000, HyTest), rabbit anti-ROCK2 (1:10000, Abcam), rabbit anti-ROCK1 (1:3000, Abcam), rabbit anti-RhoA (1:1000, Cell Signaling), rabbit anti-RhoC (1:1000, Cell Signaling), rabbit anti-phospho-Src Tyr 409 (1:1000, Cell Signaling), rabbit anti-Src (1:1000, Cell Signaling), rabbit anti-CSK (1:1000, Cell Signaling), mouse anti-GFAP (1:500, BD Pharmingen), mouse anti-vimentin (1:500, Thermo Scientific), rabbit anti-profilin1 (1:3000, Abcam).

For chondroitin 4 sulfate quantification cell lysates were first treated with chondroitinase ABC 0.05 UN/ml for 2 h at 37°C, as previously described (Chan et al., 2007), and then loaded 12% SDS-Page gel and transferred to nitrocellulose membranes (Amersham). Mouse anti-C4S (1:1000, Millipore) primary antibody was used.

Band intensity was quantified using a densitometer (Bio-Rad) and quantity one software, for all membranes. For a semi-quantitative evaluation of the C4S expression all bands present in each lane were quantified.

#### Förster resonance energy transfer analysis

Astrocytes plated on glass-bottom culture dishes (μ-Dish 35 mm, iBidi) and transfected with the FRET probes for RhoA (Raichu-EV-RhoA, ref pmid 12860967) or Src (KRas-Src-YPet, ref pmid 18799748) were imaged in an inverted epifluorescence microscope (DMI6000B, Leica Microsystems). The donor fluorescent protein was excited with a mercury lamp coupled to a light attenuator (EL6000, Leica Microsystems), and the emission of both donor and acceptor fluorescent proteins was acquired with a digital CMOS camera (4 × 4 binning, ORCA-Flash 4.0 V2, Hamamatsu Photonics). A 440–520 nm dichroic mirror (CG1, Leica Microsystems) was used together with appropriate emission and excitation filters mounted in external filter wheels (Fast Filter Wheels, Leica Microsystems). LAS AF software (Leica Microsystems) was used to control all modules. Raw images were background subtracted and time-lapse videos representing FRET ratio values (FRET/Donor or Donor/FRET) were generated. Regions of interest were drawn over cells and detailed analysis was performed to generate time-plots. Videos were converted to intensity modulated display mode using custom ImageJ macros and FRET channel as intensity modulator. Src kinase inhibitor (SKI-1) was added (200 nM) as a chemical inhibitor of SRC.

#### Statistical analysis

Statistical analysis was performed using the Graphpad Prism program (version 5). Statistical differences between groups were determined based on One-Way ANOVA tests followed by Tukey's post-hoc analysis (multiple comparisons) or t-student tests (2 group comparison). When Gaussian distribution was not confirmed (D'Agostino and Pearson omnibus normality analysis), non-parametric tests were applied. Man-Whitney test and Kruskal-Wallis test followed by the Dunn's multiple comparison test were used in the case of paired and multiple comparisons, respectively. Data are expressed as the mean ± standard deviation and *p* < 0.05 were considered significant.

## Results

### Meningeal fibroblasts conditioned medium mimics fibroblast infiltration and activates astrocytes

**Figure 1:**
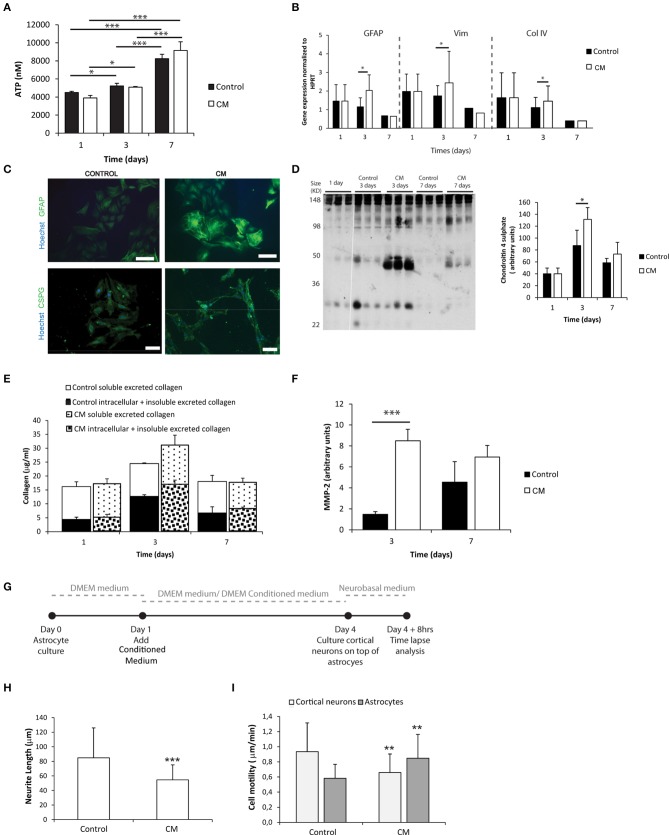
**Astrocyte 2D culture in the presence or absence of meningeal fibroblasts conditioned medium (CM)**. Results are shown as mean ± standard deviation; asterisks represent statistical differences, ^*^p < 0.05, ^**^p < 0.01, ^***^p < 0.001 **(A)** ATP levels at 1, 3, and 7 days of culture (*n* = 6, statistical analysis was performed between all pairs of columns); **(B)** mRNA levels of astrocytes at 1, 3, and 7 days of culture (*n* = 6, statistical analysis was performed between control and CM conditions at each time point); **(C)** Immunocytochemistry of astrocytes cultured in the presence of CM and control conditions, scale bar 100 μm; **(D)** Quantification of Chondroitin 4 sulfate (C4S) at 1, 3, and 7 days of culture. For each time point 3 samples from independent experiments were loaded in the gel. Quantification graph refers to whole lane (*n* = 3, statistical analysis was performed between control and CM conditions at each time point); **(E)** Collagen quantification during the cell culture time. Collagen was measured in the supernatant (collagen excreted to the culture medium) and in the culture well (Deposited and cytoplasmic collagen) (*n* = 3; no statistical differences were found); **(F)** MMP expression levels (*n* = 3, statistical analysis was performed between control and CM conditions at each time point). Effect of control and CM treated astrocytes on cortical neurons; **(G)** Experimental set-up. Astrocytes were cultured for 4 days with fresh medium or conditioned medium. At day 4 cortical neurons were cultured on top of the astrocytes for additional 4 days; **(H)** Effect of astrocytes on axonal outgrowth (*n* = 95 cells); **(I)** Cell migration velocity (*n* = 30 cells).

Astrocytes were cultured in the presence of meningeal fibroblasts conditioned medium (CM). The metabolic activity of astrocytes increased with culture time in both control and CM conditions (Figure 1A). Astrocytes cultured in the presence of CM showed increased expression of astrogliosis hallmark genes as *Gfap* and *Vimentin* (Figure 1B) and proteins as GFAP and C4S at 3 days when compared to controls (Figures 1C,D). Although no statistical differences were found between control and CM, total collagen expression levels, both intracellular and deposited non-soluble collagen, also peaked upon 3 days of culture (Figure 1E). Additionally, astrocytes cultured with CM exhibited significantly increased levels of excreted active MMP-2 upon 3 days of culture compared with controls (Figure 1F). When neurons were co-cultured with astrocytes, which had been previously cultured in CM (Figure 1G), neurite length was significantly impaired (Figure 1H). Moreover, the motility of these neurons was diminished whereas astrocyte motility increased (Figure 1I).

### Astrocytes can be successfully cultured within 3D alginate matrices

**Figure 2:**
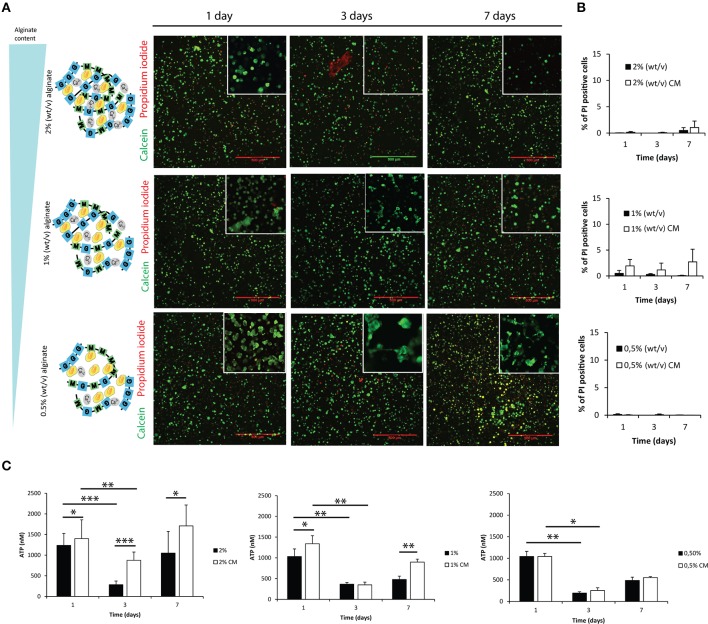
**Evaluation of cell viability and metabolic activity within 3D alginate discs**. Results are shown as mean ± standard deviation; asterisks represent statistical differences ^*^p < 0.05, ^**^p < 0.01, ^***^p < 0.001 **(A)** Representative photos of live-dead assay for astrocytes cultured in the presence of fibroblast conditioned medium (CM). Live cells are stained with Calcein-AM (green) and dead cells are stained with propidium iodide (red); **(B)** Quantification of dead cells (propidium iodide) by FACS analysis at 1, 3, and 7 days of culture (*n* = 3 pools of 10000 events); **(C)** ATP levels of the 3D culture astrocytes at 1, 3, and 7 days of culture (*n* = 6).

Astrocytes were cultured within alginate hydrogel discs of different alginate content as illustrated in Figure 2A, Image 1, namely 2, 1 and 0.5% alginate. Astrocytes remained viable throughout the 7-day culture period (Figures 2A,B). ATP consumption levels varied in time, deepening at the third day of culture to recover initial values at day 7 (Figure 2C). Such reduction in ATP levels was not the result of a decrease in cell viability, as flow cytometry analysis showed that independently of the culture time and tested conditions, 95% of cells were propidium iodide negative (Figure 2B).

### 3D cultured astrocytes acquire a reactive-like phenotype

**Figure 3:**
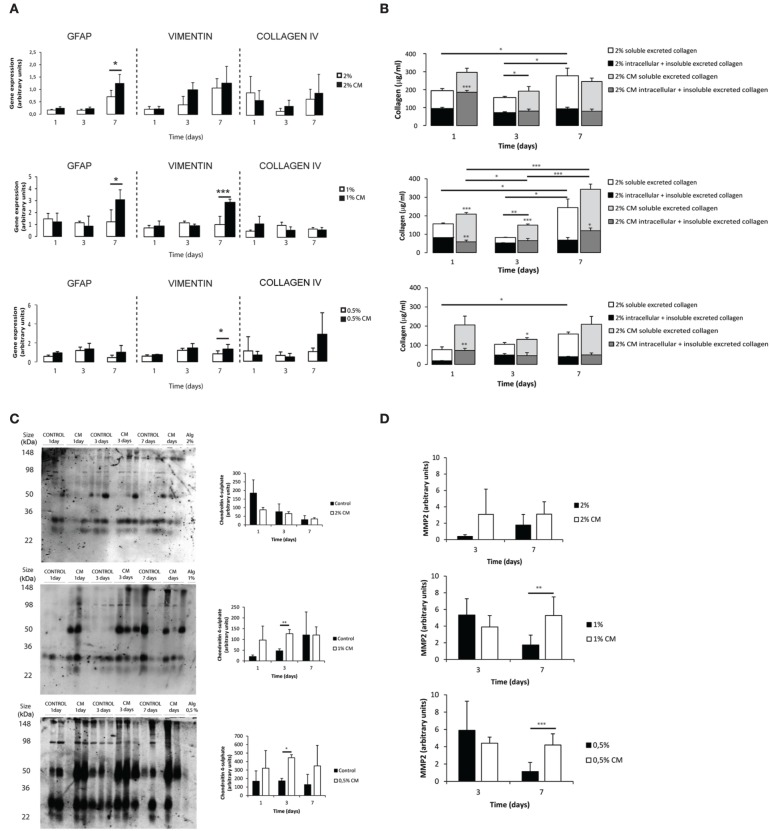
**Phenotype and matrix production of 3D seeded astrocytes**. Results are shown as mean ± standard deviation; asterisks represent statistical differences, ^*^p < 0.05, ^**^p < 0.01, ^***^p < 0.001 **(A)** mRNA levels for Gfap, Vimentin and Collagen IV of astrocytes seeded within alginate discs; **(B)** Collagen quantification at 1, 3, and 7 days of culture. Collagen was measured in the supernatant (collagen excreted to the culture medium) and in the alginate disc (Deposited and cytoplasmic collagen) (*n* = 3), regarding statistical analysis asterisks alone represent differences between of one component (excreted or intracellular collagen) and the control, asterisks above the bars with guidance line represent differences between total collagen levels; **(C)** Quantification of chondroitin 4 sulfate (C4S) at 1, 3, and 7 days of culture. For each time point 3 samples from independent experiments were loaded in the gel. Quantification graphs refer to whole lane (*n* = 3); **(D)** MMP-2 activity at 3 and 7 days of culture (*n* = 4).

Astrocytes cultured within alginate matrices show different gene expression levels when cultured in the presence or absence of meningeal fibroblasts CM (Figure 3A). Particularly, *Gfap* and *Vimentin* levels are differently regulated after 7 days of culture. Only astrocytes cultured within 1% alginate discs showed increased expression of both *Gfap* and *Vimentin* when cultured in CM. Although *Collagen IV* mRNA expression levels were not significantly different between CM and control culture conditions (Figure 3A), the presence of CM induced astrocytes to increase collagen excretion levels at 1 and 3 days of culture (Figure 3B). At day 7, significant differences were only seen on astrocytes cultured in 1% alginate discs. Moreover, collagen levels were found to be 10 times higher in all alginate formulations than those found for 2D cultured astrocytes (Figures 1E, 3B). Additionally, alginate content appears to affect collagen production levels, with cells cultured in gels with a higher alginate content producing, in general, more collagen than those seeded in gels with a lower alginate content. Nevertheless, it is important to say that this is not true for astrocytes seeded in 1% alginate discs in the presence of CM upon 7 days of culture, as these present higher levels of collagen production than the ones cultured in 2% alginate discs in the presence of CM. Furthermore, the presence of CM also induces 3D cultured astrocytes to produce higher levels of CSPG, with a significant increase of CSPG levels at day 3 of culture (Figure 3C). No significant differences were found between 2D and 3D conditions CSPG levels (Image 2). Upon 7 days of culture MMP-2 expression levels were also found to be upregulated in astrocyte cultures in 1% and 0.5% (Figure 3D).

**Figure 4:**
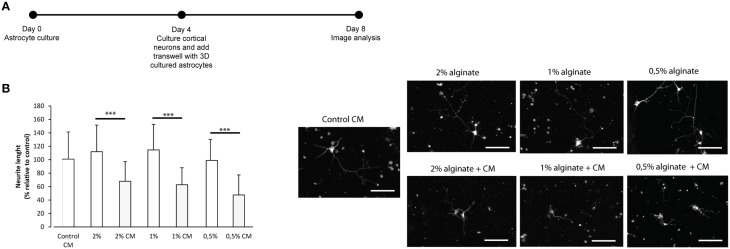
**Effect of control and CM treated astrocytes seeded in 3D alginate discs on cortical neurons**. Results are shown as mean ± standard deviation; asterisks represent statistical differences ^***^p < 0.001 **(A)** Experimental set-up. Astrocytes were cultured for 4 days with fresh medium or conditioned medium. Neurons were cultured on glass cover-slips in 24 well-plates, alginate discs were placed on top of neurons using transwells; **(B)** (Left) Effect of astrocytes on axonal outgrowth (*n* = 95 cells); (right) immunocytochemistry of cultured cortical neurons with anti-TAU, at the day of image analysis, scale bar 100 μm.

To further confirm the reactive phenotype of the 3D cultured astrocytes, cortical neurons were co-cultured with 3D astrocyte cultures. Neurite outgrowth was impaired when neurons were co-cultured with 3D astrocyte cultures treated with CM, for all alginate formulations (Figure 4).

### 3D seeded astrocytes influence hydrogels mechanical properties

The calculated mesh size of the hydrogels was found to be dependent on alginate content. The 2% alginate discs showed a smaller mesh size than 1% alginate discs and 0.5% alginate discs presented the highest mesh size (Image 3).

**Figure 5:**
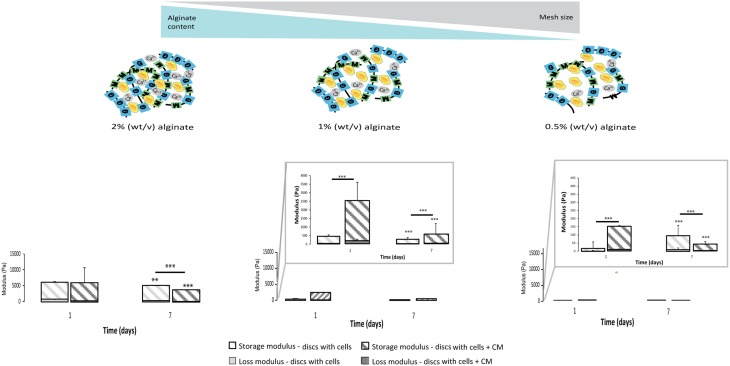
**Physical properties of the 3D alginate discs**. Rheological properties of the 3D alginate discs incubated with culture medium or with astrocytes in the presence or absence of CM (*n* = 3). Results are shown as mean ± standard deviation; asterisks represent statistical differences,), regarding statistical analysis asterisks alone represent differences between storage modulus, asterisks above the bars with guidance line represent differences between total levels, ^**^p < 0.01, ^***^p < 0.001.

Mechanical properties of alginate discs with and without astrocytes were analyzed by rheometry. Alginate hydrogels presented typical mechanical spectra of gels with a solid-like character (G′- storage modulus) predominant over liquid-like viscous response (G″- loss modulus). Moreover, the mechanical properties of hydrogel discs varied in an alginate content dependent manner (Image 3). A 10-fold difference in stiffness was observed between consecutive alginate formulations (from 2% to 1%, and from the latter to 0.5%, Image 3, Figure 5).

Astrocytes cultured in the presence of CM were able to dynamically alter their original mechanical environment and reinforce the overall disc mechanical properties. This stiffening of the storage modulus was particularly clear for astrocytes cultured with CM in 1% alginate discs at 24 h (Figure 5), with statistically higher storage modulus values than those of discs with astrocytes and of discs without cells. In general, storage modulus values of discs with astrocytes, cultured with or without CM, decreased from day 1 to day 7.

### The rho-rock signaling pathway regulates astrocyte reactivity

The Rho-ROCK signaling pathway was investigated as a possible mediator of astrocyte activation and consequent production of inhibitory molecules. Small hairpin RNAs were initially used to knockdown several members of the Rho family (*RhoA, RhoB*, and *RhoC*). Knockdowns were validated in astrocytes by qPCR, with consistent high gene-knockdown (superior to 70%) being achieved (Image 4). *RhoA* and *RhoC* knockdowns had a significant influence on *Gfap* expression in astrocytes, as *RhoA* knockdown resulted in decreased levels of *Gfap*, and *RhoC* knockdown in increased *Gfap* levels. Moreover, *RhoA* and *RhoC* knockdown also caused alteration in *Src* gene expression (Image 4).

**Figure 6:**
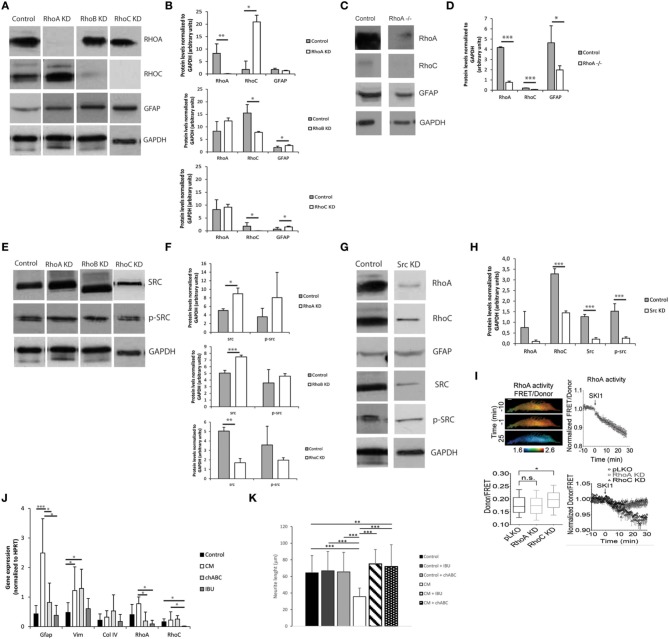
**The Rho-ROCK pathway influence on astrocyte reactivity**. Results are shown as mean ± standard deviation; statistical analysis was performed between conditions at each time point, ^*^p < 0.05, ^**^p < 0.01, ^***^p < 0.001 **(A)** Representative images of western blots of RHOA, RHOC, and GFAP levels on RhoA, RhoB, and RhoC knockdown astrocytes; **(B)** Western blot quantification (*n* = 3); **(C)** Representative images of western blot of lox/lox RhoA -/-; **(D)** Western blot quantification (*n* = 3); **(E)** Representative images of western blot of c-SRC levels on RhoA, RhoB, and RhoC knockdown astrocytes; **(F)** Western blot quantification (*n* = 3); **(G)** Representative images of western blot from Src Knockdown astrocytes; **(H)** Western blot quantification (*n* = 3); **(I)** FRET analysis of RHOA and SRC activity in astrocytes. SKI-1 was used as a chemical inhibitor of SRC; **(J)** Effect of the treatment of reactive astrocytes with 0.1 U/ml chABC and 0,04M ibuprofen. Astrocytes were cultured for 7 days in the presence of CM and then treated with chABC or Ibuprofen for 48 h. (KD stands for knockdown); **(K)** Effect of reactive astrocytes treated with chABC or ibuprofen (IBU) on cortical neurons. Neurons were cultured in glass coverslips and alginate discs with astrocytes were placed on top using transwells.

Knockdown influence on protein expression was determined by western blot (Figures 6A,B). All knockdowns resulted in significantly reduced levels of protein, with RHOA and RHOC protein levels decreased by 99%. Western blot analysis confirmed that *RhoA* and *RhoC* knockdown and respective proteins differently affect GFAP protein levels (Figures 6A–D), with *RhoA* knockdown resulting in down-regulation of GFAP protein levels and *RhoC* and *RhoB* knockdown in up-regulation of those levels. The effect of RhoA in GFAP levels was further confirmed following the ablation of RhoA in RhoA^lox/lox^ astrocytes (Figures 6C,D).

### c-SRC regulates RhoA activity

The src family has been shown to cross-talk with Rho during intracellular signaling, src kinase is further known to bind and phosphorylate RhoGDI both *in vitro* an *in vivo* at Tyr 156 (Belsches et al., 1997; DerMardirossian et al., 2006).

*RhoA* and *RhoB* knockdowns resulted in up-regulation of c-SRC while *RhoC* knockdown resulted in SRC down-regulation (Figures 6E,F). *c-src* knockdown not only resulted in c-SRC downregulation but also impacted the levels of total Src family tyrosine kinase (SFK) activity as demonstrated by the reduction in Tyrosine 416 phosphorylated SFK levels (Figures 6G,H).

Knocking down *c-src* also resulted in down-regulation of RHOA and RHOC protein levels. To investigate whether SRC regulates RHOA activation, we measured RHOA activity in astrocytes using a Förster Resonance Energy Transfer (FRET)-based sensor for RHOA with enhanced sensitivity, and show that inhibition of c-SRC by the SRC pharmacological inhibitor Ski-1 resulted in decreased RHOA activity (Figure 6I). In contrast, knocking down *RhoA* did not significantly influence c-SRC activity, as measured by a FRET-based sensor for c-SRC. Interestingly, c-SRC activity was significantly increased in RHOC knockdown astrocytes (Figure 6I).

### Astrocyte reactivity is reverted via RhoA inhibition

Astrocytes were cultured within 1% alginate discs in the presence of CM, and after 7 days of culture chondroitinase ABC (chABC) or ibuprofen (IBU) were added to the culture medium. Activated astrocytes treated with chABC showed significantly lower *RhoA* levels than those untreated (CM-activated), and comparable to the control levels (Figure 6J). *Gfap* expression levels followed a similar trend. Nevertheless, *Vimentin* levels were not different from those of CM-treated astrocytes and were statistically higher than those of control cells. Regarding cells treated with ibuprofen, *RhoA* levels were also significantly reduced when compared to CM-treated astrocytes, to levels comparable to control cells. *Gfap* and *Vimentin* expression levels were also significantly reduced, when compared to CM-treated cells, and were at comparable levels of the control. *RhoC* levels were also significantly reduced in comparison to all tested conditions (Figure 6J).

In order to further confirm astrocyte phenotype recovery, astrocytes cultured within 1% alginate discs treated with chABC and IBU were co-cultured with cortical neurons (Figure 6K). Neurite outgrowth was not impaired when neurons were co-cultured with 3D cultured astrocytes treated with chABC or IBU (Figure 6K).

## Discussion

A tissue-engineered astrogliosis 3D-culture model is of added value. It is more physiologically relevant than the existing 2D models, as it can recapitulate better cellular interactions *in vivo*. This is particularly significant when considering astrocytes, which are known to form a 3D cellular network that extends throughout the brain (Mathewson and Berry, 1985). The role of this network is not well understood yet; however, it likely plays a pivotal role in astrocytic response to injury, since most brain pathologies result in some degree of deformation of this structure (Ostrow and Sachs, 2005). Alginate hydrogels are an attractive choice for such models for several reasons: firstly because of their mechanical properties, which recapitulate the brain's mechanical properties (Banerjee et al., 2009); secondly, they work as an inert backbone structure that allow the control over system's complexity (Rowley et al., 1999; Lutolf and Blau, 2009); finally, an extremely relevant feature is that the cross-linking of these hydrogels can be reversed with the use of quelators (e.g., EDTA), enabling the recovery of the cultured cells for further biochemical and cellular assays. Here, the glial scar environment was closely recreated by stimulating astrocytes culture in alginate hydrogels with tuned mechanical properties with fibroblast CM.

Astrocyte activation with CM, and their consequent ability to inhibit axonal outgrowth, was validated in 2D astrocytic cultures (Figure 1). To the best of our knowledge meningeal fibroblast's CM has never been used to induce astrocyte reactivity. Some authors have previously explored co-cultures of cerebral astrocytes and meningeal fibroblasts (Abnet et al., 1991; Struckhoff, 1995; Hirsch and Bähr, 1999; Wanner et al., 2008; Kimura-Kuroda et al., 2010), nevertheless, the use of CM enables an increased control over the model system variables.

Given the mild gelation conditions, astrocytes remain viable when cultured within the tested alginate hydrogels (Figures 2A,B). The reduction of ATP levels at 3 days of culture may be explained by an initial adaptation phase of astrocytes to the new surrounding environment. Reactive astrocytes are known to have increased metabolic activity (Zamanian et al., 2012), and astrocytes cultured in the presence of CM showed increased activity in 2 and 1% alginate discs when compared to control (Figure 2C), which was not observed in 2D astrocytic cultures (Figure 1A). The presence of CM significantly induced an increase in expression levels of *Gfap* and *Vimentin* (Figures 1B, 3A) in cultured astrocytes. Intermediate filaments augmentation is widely correlated with astrogliosis and glial scaring (Wang et al., 2004; Ogier et al., 2005; Nair et al., 2008; Wanner et al., 2008; Kimura-Kuroda et al., 2010; Middeldorp and Hol, 2011).

Increased deposition of ECM molecules and their interaction with local cells is considered an important factor in the non-permissive nature for CNS repair (Sobel and Mitchell, 1989; van Horssen et al., 2007). For this reason, ECM production was assessed. Increased CSPGs production is also a hallmark of astroglisosis and is known to play a pivotal role in neurite outgrowth inhibition (Jones et al., 2003; Galtrey and Fawcett, 2007). In fact, astrocytes seeded in the presence of CM have shown increased CSPGs production levels (Figures 1C,D, 3C) and were further shown to inhibit neuronal outgrowth (Figures 1H, 4B). This is particularly relevant, as many authors have considered cultured astrocytes too immature to mimic reactive astrogliosis because they would promote rather than inhibit neurite outgrowth (Fallon, 1985; Smith et al., 1990). In addition to the CSPG analysis already mentioned, collagen levels were also assessed. Interestingly, collagen production was 10 times higher in 3D cultured astrocytes than in 2D (Figures 1E, 3B). The increased collagen production in 3D astrocyte cultures in comparison to the 2D cultures, suggests an important role of the 3D structure in regulating ECM production. Within the 3D culture, collagen levels were significantly increased by the presence of CM in astrocytes cultured within 2 and 1% discs.

MMPs are known to be up-regulated in reactive astrocytes under pathological conditions (Rivera et al., 1997; Rathke-Hartlieb et al., 2000). In addition, MMP-2 (a type IV collagenase) was shown to be the most active enzyme in the degradation of myelin basic protein (MBP) (Chandler et al., 1995). Increased MMP-2 activity was seen in 2D cultures at 3 days of culture (Figure 1F). In the established 3D systems, MMP-2 activity was found to be indirectly proportional to the alginate content (Figure 3D). Moreover, upon 7 days of culture, astrocytes cultured within 1 and 0.5% alginate in the presence of CM showed significantly increased MMP-2 activity.

Overall, astrocytes cultured in CM within 1% alginate discs resembled more closely scar astrocytes as these showed increased gene expression levels of *Gfap* and *Vimentin* (Figure 3A), as well as increased ECM production (Figures 3B,C). However, as the CM stimulus was present in all tested alginate formulations, astrocytes were probably capable of sensing and responding to the physical microenvironment they were in, as this was the only variable of the systems. Therefore, to further elucidate the differences in matrix stiffness of the different alginate formulations under study, rheological studies were performed and mesh size was estimated.

Calculated mesh size varied slightly, within the nanometer range, in the three tested alginate formulations with values below the cellular size (Image 3) so, it can be considered that it was possible to change the matrices mechanical properties independently of the mesh size. The prepared hydrogels showed a viscoelastic behavior, typically observed in ECM-derived hydrogels (Bott et al., 2010). As expected, the discs with higher alginate content showed higher stiffness values (Image 3). Furthermore, it was possible to observe that astrocytes in softer alginate matrices (1 and 0.5%) effectively stiffened the hydrogel after 24 h of culture with CM (Figure 5). This effect was particularly evident for astrocytes cultured within 1% alginate matrices, which can be correlated with the increased production of collagen and/or CSPG (Figures 3B,C). As cellular behavior was significantly affected by the mechanical properties of the alginate 3D matrices, the only variable parameter, we hypothesized that astrocyte activation is mediated by a mechanosensing pathway.

The Rho/ROCK signaling pathway is known to play a critical role in the assembly of actin stress fibers in response to applied mechanical forces (Aikawa et al., 1999; Putnam et al., 2003). Moreover, the small GTPase Rho is a key regulator of intracellular contractility allowing cells to sense matrix stiffness and respond to mechanical cues (Wozniak et al., 2003). Taking this in consideration, the Rho/ROCK signaling pathway was explored as a possible mediator of astrocytic activation.

Data from qPCR and western blot have shown that *RhoA* and *RhoC* differently regulate *Gfap* expression, with *RhoA* knockdown decreasing *Gfap* expression while *RhoC* knockdown up-regulates it (Image 4, Figure 6). As such, *RhoA* is here promoting *Gfap* expression while *RhoC* is blocking it. Western blot data suggests that c-SRC regulation is closely correlated with RHOA (Figure 6). FRET analysis showed that c-SRC is positively regulating RHOA activation levels, as chemical inhibition of SRC with SKI-1 in astrocytes induced a decrease in RHOA activity. Furthermore, these results show that in astrocytes c-SRC is responsible for part of the SFK activity.

RHOA has previously been shown to play a role in focal adhesion formation in astrocytes (Matthews et al., 2006; Khatiwala et al., 2009), and inhibition of the Rho/ROCK signaling pathway was shown to increase astrocyte reactivity (Chan et al., 2007; Lau et al., 2011); nonetheless, so far to the best of our knowledge no one has shown the individual influence of RHOA and RHOC on GFAP expression in astrocytes. This makes RHOA an interesting therapeutic target for astrogliosis treatment.

To further assess the role of Rho/ROCK signaling in the 3D culture model, astrocytes were cultured in the presence of two drugs known to affect RhoA: ibuprofen and chABC. Non-steroidal anti-inflammatory drug Ibuprofen was previously shown to effectively block *RhoA* (Wang et al., 2009; Dill et al., 2010), and the latter is known to mediate the inhibition of axonal regeneration by myelin and CSPGs (Yiu and He, 2006; Walker et al., 2013). Although, *RhoA* has been mostly studied in neurons, our data suggests that it can be a pivotal modulator of astrocyte behavior. Astrocyte reactivity appeared to be achieved through increased *RhoA* levels and not *RhoC*'s reduction. Moreover, analysis of gene expression levels revealed that treatment with Ibuprofen or chABC had an impact on *RhoA* levels as those of treated cells were significantly lower than those of reactive astrocytes. Moreover, *Gfap* levels were also significantly reduced to levels comparable to the control (Figure 6J). Although, ibuprofen and chABC are known to target different molecules they both inhibited RhoA. Ibuprofen is known to directly inactivate RhoA while chABC is known to degrade the ECM. These results reinforce the idea that ECM remodeling is a cause of astrocyte reactivity and, as such, when ECM is degraded by chABC astrocyte phenotype is recovered. As such, RhoA inhibition on reactive astrocytes, either through chABC or ibuprofen treatment, generated an environment permissive to neurite outgrowth.

Overall, this work established the potential of a glial scar like hydrogel based 3D model. Cells in this culture model behaved similarly to scar astrocytes, showing changes in gene expression and increased ECM production leading to neuronal outgrowth inhibition. Moreover, exploring the mechanisms regulating astrogliosis showed a pivotal role of *RhoA* in astrocyte reactivity. As such, RhoA is here seen as a therapeutical target while Ibuprofen and chABC are explored as possible approaches to diminish astrogliosis.

This simple, controlled and reproducible 3D culture system constitutes a good scar-like system and offers great potential in future neurodegenerative mechanism studies, as well as in drug screenings envisaging the development of new therapeutic approaches.

### Conflict of interest statement

The authors declare that the research was conducted in the absence of any commercial or financial relationships that could be construed as a potential conflict of interest.

## Supplementary material

Image 1 **Evaluation of cell viability of 3D cultured control astrocytes**. Representative photos of live-dead assay. Live cells are stained with Calcein-AM (green) and dead cells are stained with propidium iodide (red).

Image 2 **Quantification of chondroitin 4 sulfate (CS4) of astrocyte cultures treated with fibroblast conditioned medium (CM) with the highest levels for each tested condition**. For each time point 3 samples from independent experiments were loaded in the gel. Quantification graph refer to whole lane (*n* = 3; mean ± standard deviation; no statistical differences were found).

Image 3 **Physical properties of the alginate discs**. **(A)** Calculated mesh size of the tested alginate formulations; **(B)** Rheological properties of alginate discs without astrocytes.

Image 4 **qPCR data for astrocyte knockdowns**. Data is presented as mean ± standard deviation, statistical analysis was performed in relation to control samples, statistical comparisons were performed between conditions at each time point, ^*^*p* < 0.05, ^**^*p* < 0.01, ^***^*p* < 0.001 **(A)** RhoA knockdown astrocytes; **(B)** RhoB knockdown astrocytes; **(C)** RhoC knockdown astrocytes; **(D)** Src knockdown astrocytes.
