# Supplementary material for: Extracellular environment contribution to astrogliosis—lessons learned from a tissue engineered 3D model of the glial scar
Source: Front Cell Neurosci. 2015 Sep 29;9:377. doi: 10.3389/fncel.2015.00377 (PMC4586948; doi:10.3389/fncel.2015.00377)
Supplement: Image 1 — Evaluation of cell viability of 3D cultured control astrocytes. Representative photos of live-dead assay. Live cells are stained with Calcein-AM (green) and dead cells are stained with propidium iodide (red). [file Image1.PDF]

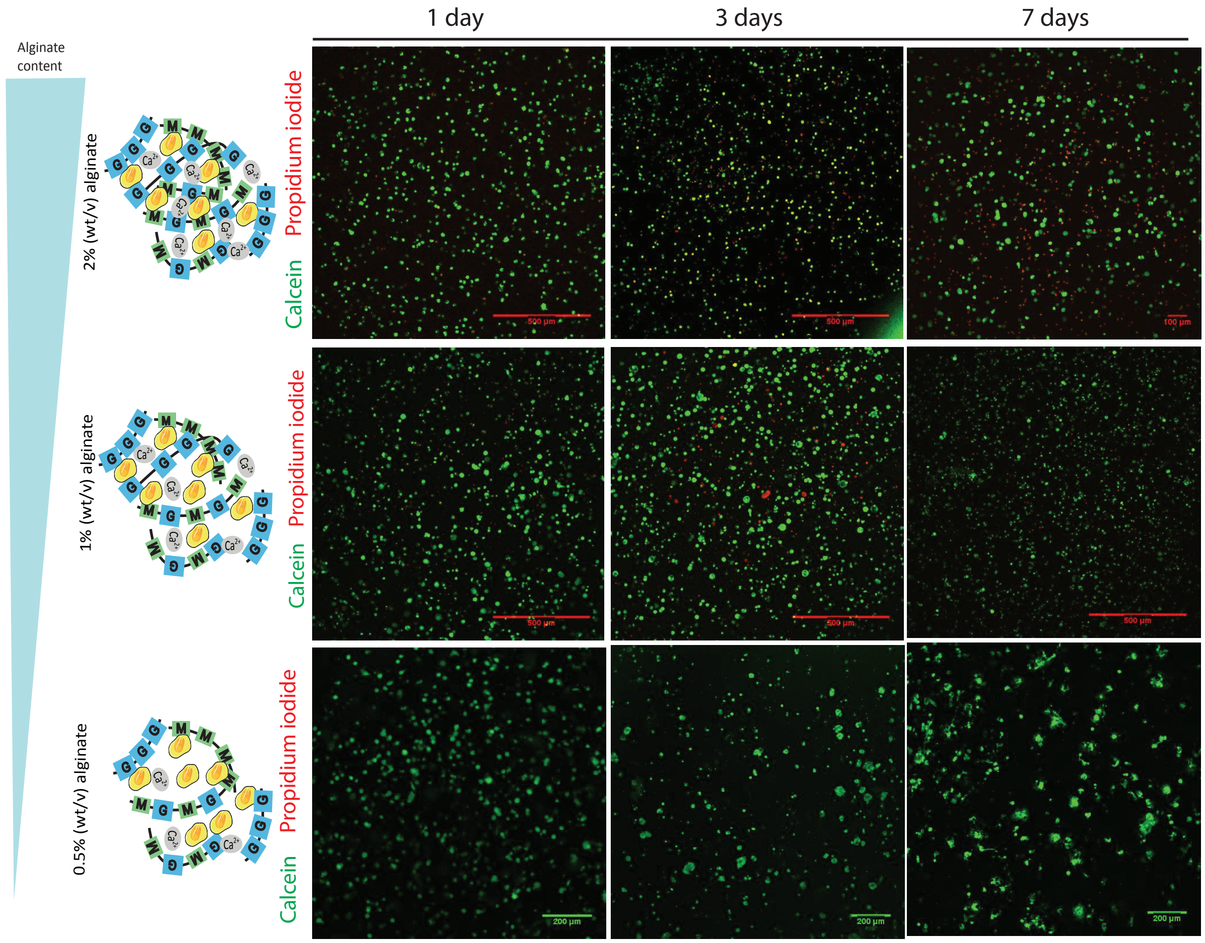
1 day
3 days
7 days
Alignate
content
2% (wt/v) alginate
1% (wt/v) alginate
0.5% (wt/v) alginate
Calcein
Propidium iodide
500 µm
100 µm
200 µm
