# Supplementary figures and images for: Extracellular environment contribution to astrogliosis—lessons learned from a tissue engineered 3D model of the glial scar
Source: Front Cell Neurosci. 2015 Sep 29;9:377. doi: 10.3389/fncel.2015.00377 (PMC4586948; doi:10.3389/fncel.2015.00377)

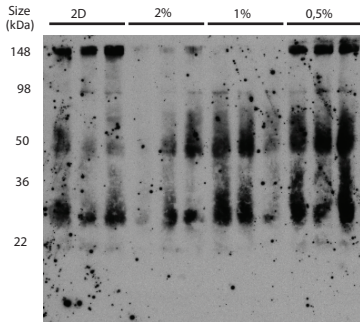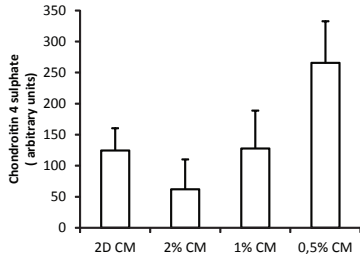

Supplement: Image 2 — Quantification of chondroitin 4 sulfate (CS4) of astrocyte cultures treated with fibroblast conditioned medium (CM) with the highest levels for each tested condition. For each time point 3 samples from independent experiments were loaded in the gel. Quantification graph refer to whole lane (n = 3; mean ± standard deviation; no statistical differences were found). [file Image2.PDF]

A

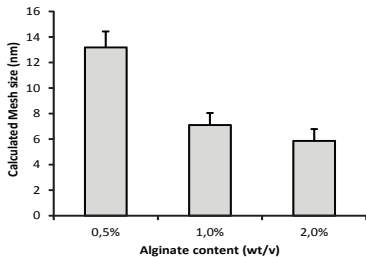

B

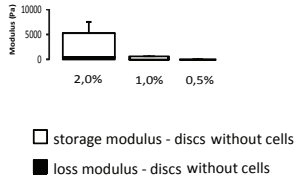

Supplement: Image 3 — Physical properties of the alginate discs. (A) Calculated mesh size of the tested alginate formulations; (B) Rheological properties of alginate discs without astrocytes. [file Image3.PDF]

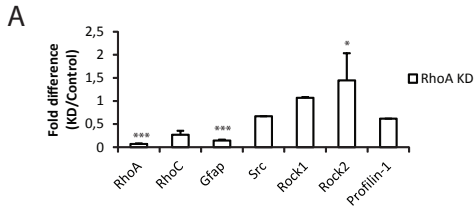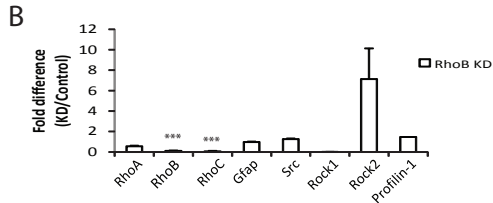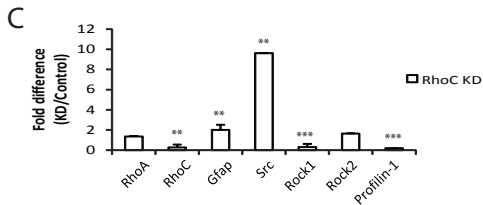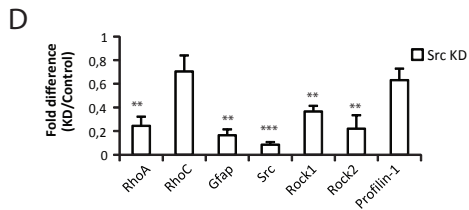

Supplement: Image 4 — qPCR data for astrocyte knockdowns. Data is presented as mean ± standard deviation, statistical analysis was performed in relation to control samples, statistical comparisons were performed between conditions at each time point, *p < 0.05, **p < 0.01, ***p < 0.001 (A) RhoA knockdown astrocytes; (B) RhoB knockdown astrocytes; (C) RhoC knockdown astrocytes; (D) Src knockdown astrocytes. [file Image4.PDF]
